# Transcriptome Analysis of *Portunus trituberculatus* in Response to Salinity Stress Provides Insights into the Molecular Basis of Osmoregulation

**DOI:** 10.1371/journal.pone.0082155

**Published:** 2013-12-03

**Authors:** Jianjian Lv, Ping Liu, Yu Wang, Baoquan Gao, Ping Chen, Jian Li

**Affiliations:** 1 Key Laboratory for Sustainable Utilization of Marine Fisheries Resources, Ministry of Agriculture, Yellow Sea Fisheries Research Institute, Chinese Academy of Fishery Sciences, Qingdao, China; 2 College of Fisheries and Life Science, Shanghai Ocean University, Shanghai, China; NIGMS, NIH, United States of America

## Abstract

**Background:**

The swimming crab, *Portunus trituberculatus*, which is naturally distributed in the coastal waters of Asia-Pacific countries, is an important farmed species in China. Salinity is one of the most important abiotic factors that influence not only the distribution and abundance of crustaceans, it is also an important factor for artificial propagation of the crab. To better understand the interaction between salinity stress and osmoregulation, we performed a transcriptome analysis in the gills of *Portunus trituberculatus* challenged with salinity stress, using the Illumina Deep Sequencing technology.

**Results:**

We obtained 27,696,835, 28,268,353 and 33,901,271 qualified Illumina read pairs from low salinity challenged (LC), non-challenged (NC), and high salinity challenged (HC) *Portunus trituberculatus* cDNA libraries, respectively. The overall de novo assembly of cDNA sequence data generated 94,511 unigenes, with an average length of 644 bp. Comparative genomic analysis revealed that 1,705 genes differentially expressed in salinity stress compared to the controls, including 615 and 1,516 unigenes in NC vs LC and NC vs HC respectively. GO functional enrichment analysis results showed some differentially expressed genes were involved in crucial processes related to osmoregulation, such as ion transport processes, amino acid metabolism and synthesis processes, proteolysis process and chitin metabolic process.

**Conclusion:**

This work represents the first report of the utilization of the next generation sequencing techniques for transcriptome analysis in *Portunus trituberculatus* and provides valuable information on salinity adaptation mechanism. Results reveal a substantial number of genes modified by salinity stress and a few important salinity acclimation pathways, which will serve as an invaluable resource for revealing the molecular basis of osmoregulation in *Portunus trituberculatus*. In addition, the most comprehensive sequences of transcripts reported in this study provide a rich source for identification of novel genes in the crab.

## Introduction

*Portunus trituberculatus* (Crustacea: Decapoda: Brachyura), commonly known as the swimming crab, is widely distributed in the coastal waters of Korea, Japan, China, and southeast Asia [1]. This species inhabits estuaries and coastal waters, which belong to typical euryhaline crab species. In China, it is a major edible crab species and one of the most important fishery resources [2] and the production has now reached 90,000 tons, valued at AUS$2.5 billion in 2009 [3].

salinity is one of the most important abiotic factors that influence not only the distribution and abundance of crustaceans, but also their general physiology and well being [4].The water salinity condition is also an important factor for artificial propagation of the swimming crab [5]. Throughout their prolonged culture period, *Portunus trituberculatus* often experience substantial salinity fluctuations either due to heavy rainfalls or droughts, which could have significant impacts to farm productivity and in severe situations, heavy mortality. This often requires the ability to regulate hemolymph osmolytes in relation to the environment they inhabit via osmoregulation to control their hemolymph osmotic pressure [6]. 

Due to the implications and critical importance of osmoregulation to the crab artificial propagation, a number of researchers have been devoted to this topic. An extensive literature that describes the growth, development, physiology, behavior, and propagation techniques of *Portunus trituberculatus* exposed to salinity stress have revealed the crab grows in optimal salinity ranged from 20-35ppt, whereas they can occur at salinities below 6 ppt and will survive salinities in excess of 48ppt [7-12]. In order to study the mechanism of osmoregulation, Xu et.al. investigated gene expression in the *Portunus trituberculatus* exposed to different salinity stresses via cDNA microarray chip, and 417 differentially expressed genes were identified [5]. Their study revealed a few important salinity acclimation pathways, which may be helpful in understanding the molecular basis of osmoregulation and salinity adaptation in the crab.

Even though cDNA microarray technology is a powerful tool for studying genome-wide gene expression, this technology fails to detect sequence variation and to recognize new genes or transcripts and can only be designed from limited expressed sequence tag data as the genome of *Portunus trituberculatus* has not yet been determined. To date, there are 13,985 ESTs available for the crab in the Genebank, however, it remains insufficient for the comprehensive understanding of *Portunus trituberculatus* transcriptome. Many low expression transcripts would be missed from current EST data, which makes it difficult for further analysis on transcriptome.

Next-generation high-throughput RNA sequencing technology (RNA-seq) is a recently-developed method for discovering, profiling, and quantifying RNA transcripts with several advantages over other expression profiling technologies including higher sensitivity and the ability to detect splicing isoforms and somatic mutations [13]. Because it is not restricted by the unavailability of a genome reference sequence, this approach has been applied in decoding the genomes of several non-model organisms, providing valuable information in the understanding of gene function, cell responses and evolution [14-16]. Significant progress has also been made in understanding the transcript expression of various marine crustacea by RNA-seq over the last two years, such as *Litopenaeus vannamei*, *Fenneropenaeus chinensis*, *Eriocheir sinensis* and *Macrobrachium nipponense* [17-21].The countable, almost digital, nature of RNA-seq data makes them particularly attractive for the quantitative analysis of transcript expression levels, which can give reliable measurements of transcript levels in one or more conditions [22]. However, such investigations in *Portunus trituberculatus* have not been reported.

In the present study, we examined the whole transcriptome responses to salinity stress of the *Portunus trituberculatus* for the first time using the Illumina’s sequencing technology. Considering individual monitoring of the *Portunus trituberculatus* responses to salinity stress, nine libraries (three technical replicates of each condition) were established from the gills of *Portunus trituberculatus* that exposed to optimal, low and high salinity seawater, respectively. The study aimed to compare the expression patterns of the three conditions to better understand the transcriptomic regulation in *Portunus trituberculatus* to salinity stress and identify genes involved in osmoregulation of the crab. The results of this study are an important resource for future researches on mechanism of osmoregulation for marine invertebrates.

## Materials and Methods

### Salinity Challenge Experiment and Sample Preparation

The swimming crabs, *Portunus trituberculatus* at 80 days age (5.62~11.66g in body weight),were obtained from a local farm in Qingdao, China. All the samples were acclimated in the laboratory (33 ppt, 18°C) for one week before the experiment treatment. The crabs were divided into 3 groups (90 crabs for one group) and acclimated to low salinity challenged (LC, 5 ppt), non-challenged (NC, 33 ppt), high salinity challenged (HC, 50 ppt) at 18°C. For each group, the sixth pair of gills from 9 crabs were collected after ten days and samples were stored in RNAlater (Ambion) at 4 °C over night and then at -20 °C until RNA extraction within 2 weeks.

### RNA Isolation, cDNA Library Construction and Illumina Deep Sequencing

Total RNA was isolated from each sample by trizol (Invitrogen, CA,USA). RNA degradation and contamination was monitored on 1% agarose gels. RNA purity was checked using the Nano Photometer® spectrophotometer (IMPLEN, CA, USA) . RNA concentration was measured using Qubit® RNA Assay Kit in Qubit® 2.0 Flurometer (Life Technologies, CA, USA).RNA integrity was assessed using the RNA Nano 6000 Assay Kit of the Bioanalyzer 2100 system (Agilent Technologies, CA, USA).A total amount of 3g RNA per sample was used as input material for the RNA sample preparations and all samples had RIN values above 8. Then, samples of three individuals were pooled within each group in equal amounts to generate three mixed sample. The pooling samples were then used to prepare 9 separate Illumina sequencing libraries (three technical replicates of each condition).

cDNA libraries were generated using Illumina TruSeq™ RNA Sample Preparation Kit (Illumia, San Diego, USA) following manufacturer’s recommendations and index codes were added to attribute sequences to each sample. Briefly, mRNA was purified from total RNA using poly-T oligo-attached magnetic beads. Fragmentation was carried out using divalent cations under elevated temperature in Illumina proprietary fragmentation buffer. First strand cDNA was synthesized using random oligonucleotides and SuperScript II. Second strand cDNA synthesis was subsequently performed using DNA Polymerase I and RNase H. Remaining overhangs were converted into blunt ends via exonuclease/polymerase activities and enzymes were removed. After adenylation of 3’ ends of DNA fragments, Illumina PE adapter oligonucleotides were ligated to prepare for hybridization. In order to select cDNA fragments of preferentially 200 bp in length the library fragments were purified with AMPure XP system (Beckman Coulter, Beverly, USA). DNA fragments with ligated adaptor molecules on both ends were selectively enriched using Illumina PCR Primer Cocktail in a 10 cycle PCR reaction. Products were purified (AMPure XP system) and quantified using the Agilent high sensitivity DNA assay on the Agilent Bioanalyzer 2100 system.

In the final step before sequencing, all 9 individual libraries were normalised and pooled together in a single lane on an Illumina HiSeq2000 platform and 90 bp paired-end reads were generated.

### Bioinformatic Analysis

#### Quality control

Raw data (raw reads) of fastq format were firstly processed through our self-written perl scripts. In this step, clean data (clean reads) were obtained by removing reads containing adapter, reads containing ploy-N and low quality reads from raw data. At the same time, Q20, Q30, GC-content and sequence duplication level of the clean data were calculated. All the downstream analyses were based on clean data with high quality.

#### Transcriptome assembly

The left files (read1 files) from all libraries/samples were pooled into one big left.fq file, and right files (read2 files) into one big right.fq file.Transcriptome assembly was accomplished based on the left.fq and right.fq using Trinity [23] with min_kmer_cov set to 2 and all other parameters set default.

#### Transcriptome Annotation

The unigenes were compared with the protein nonredundant database using BlastX with E values less than 1.0×10^-5^ (E values less than 1.0×10^-5^ were considered as significant) [24]. All annotated unigenes were used to determine the Clusters of Orthologous Groups of proteins (COG) term, Gene Ontology (GO) term and the Kyoto Encyclopedia of Genes and Genomes (KEGG) pathway with a cut-off E-value of 1.0×10^-5^ using BlastX.

#### Differential Expression, Cluster analysis and GO enrichment Analysis

Differential expression analysis was performed using the DEGseq (2010) R package. P value was adjusted using q value [25]. qvalue<0.005&|log2 (foldchange)|>1was set as the threshold for significantly differential expression. The three samples from each treatment were used to generate mean expression levels. Hierarchical cluster analysis is used to identify differentially expressed genes with certain patterns of expression under two different salinity challenge using Cluster 3.0 [5,26]. The settings for the calculations were as follows [5]: similarity is measured by standard correlation, and clustering method is average linkage. The genes are clustered according to similarities in expression proﬁles across three salinity conditions. GO enrichment analysis of the differentially expressed genes (DEGs) was performed using GOseq based Wallenius non-central hyper-geometric distribution[27], which can adjust for gene length bias in DEGs. 

### Real-time RT-PCR confirmation of Illumina sequencing data

To validate our Illumina sequencing data, twelve differentially expressed genes were selected for quantitative RT-PCR analysis, using the same RNA samples as for the transcriptome profiling. Primers were designed using the Primer5 software (Premier Biosoft International) (Table **S1**). RpL8 gene was selected as an internal control for qPCR analysis and the primers reference Xu’s literature [5]. First strand cDNA was synthesized from1 mg of RNA using M-MuLV reverse transcriptase (Qiagen). The qPCR reaction mixture (20 mL) consisted of 26 Power SYBR Green PCR Master mix, 0.9M each of the forward and reverse primers, and 1 mL of template cDNA. PCR amplification was performed under the following conditions: 50°C for 2 min and 95°C for 30 s, followed by 40 cycles of 95°C for 15 s and 62°C for 1 min, and a final extension at 72°C for 5 min.

## Result

### Illumina Draft Reads and Sequence Assembly

**Figure 1:**
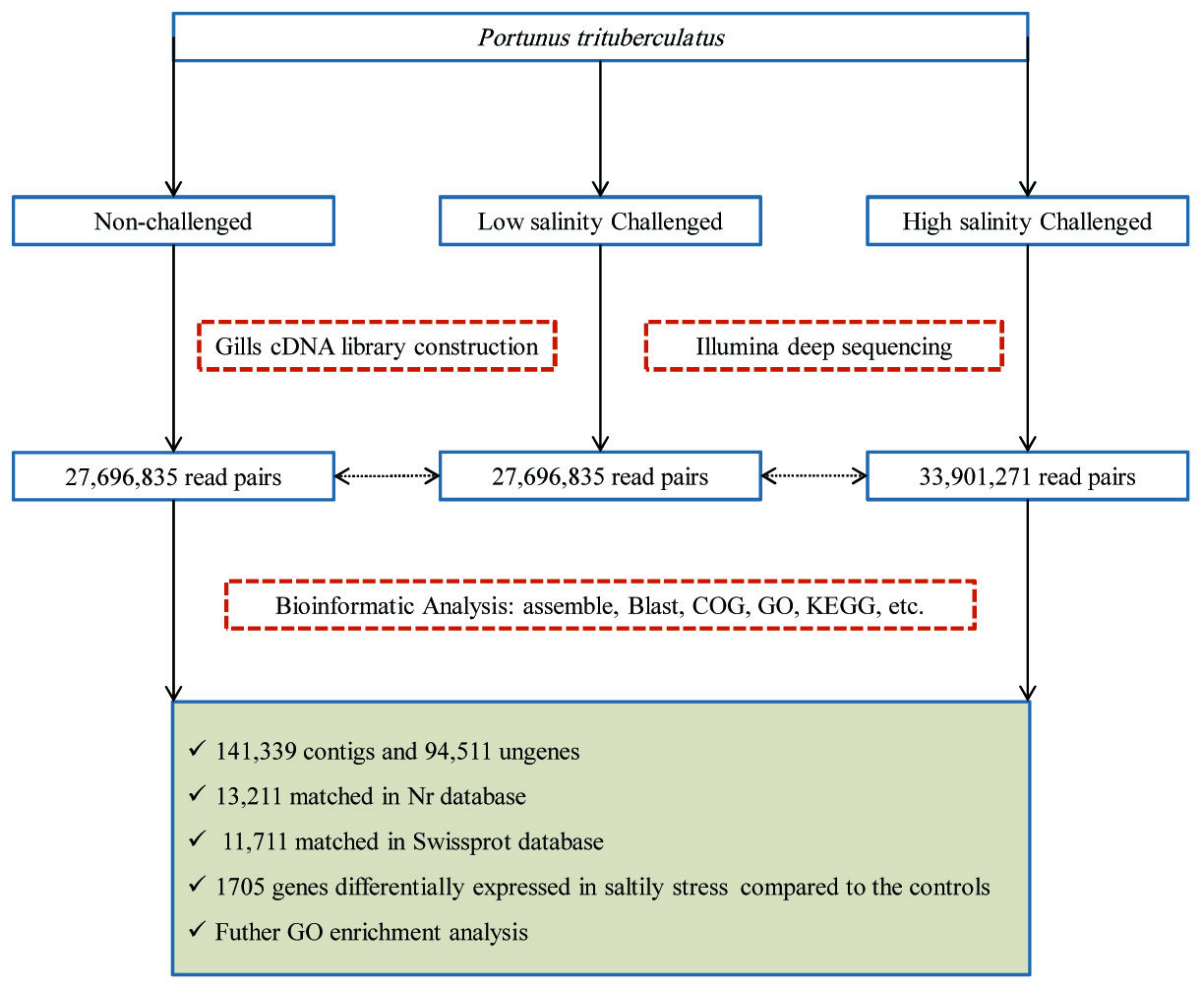
Schematic of Illumina deep sequencing and analysis. It includes sample preparation, cDNA library construction and Illumina sequencing, data analysis including assemble, blast, GO annotation, gene expression analysis, etc.

In this study, gills of three crabs were used to prepare one pooled RNA sample for each group of LC, NC and HC (three technical replicates of each group). Nine cDNA libraries were then constructed to perform Illumina sequencing. The schematic of Illumina deep sequencing and analysis are shown in Figure **1**. We obtained 27,696,835, 28,268,353, and 33,901,271 qualified Illumina read pairs for LC, NC and HC, giving rise to total clean bases of 5.60, 5.72 and 6.80G, respectively. The overall Illumina read pairs and clean bases for all samples are 89,866,459 and 18.16G, respectively (Table **1**). Files containing these data were deposited in the Short Read Archive of the National Center for Biotechnology Information (NCBI) with accession numbers of SRR1013694 (LC), SRR1013695 (NC) and SRR1013696 (HC).

**Table 1:** Summary of draft reads of three libraries by Illumina deep sequencing.

**Grou*p*^*a*^**	**PE library size (bp)**	**Read pairs**	**Read length (bp)**	**Cleanbases (G)**
**LC**	200	27,696,835	90	5.60
**NC**	200	28,268,353	90	5.72
**HC**	200	33,901,271	90	6.84
**Total**	200	89,866,459	90	18.16

a, the data of every group comes from three technical replicates

**Figure 2:**
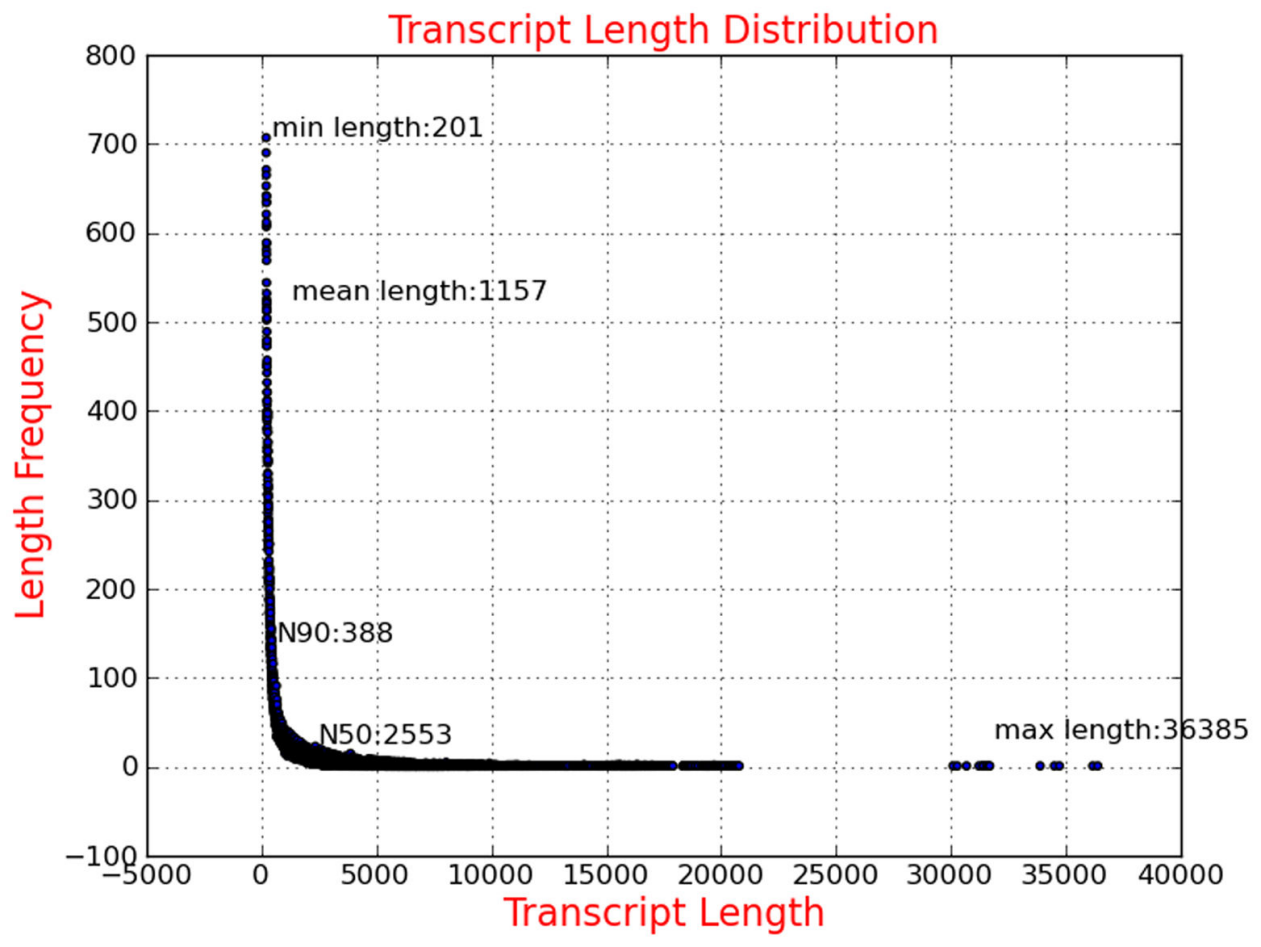
Sequence length distribution of contigs assembled from Illumina reads.

After assembly analysis based on all Illumina reads, we identified 141,339 contigs. The average length of all contigs was 1,157 bp, with the smallest sequence of 201 bp and the largest one of 36,385 bp. The sequence length distribution of contigs is indicated in Figure **2** and Table **2**. Further assembly analysis showed that all contigs contributed to 94,511 unigenes, with an average length of 644 bp. 

**Table 2:** Assembly Results Statistics.

**Transcript length interval**	**200-500bp**	**500-1kbp**	**1k-2kbp**	**>2kbp**	**total**
**Number of transcripts**	73,467	24,848	19,568	23,456	141,339

**Figure 3:**
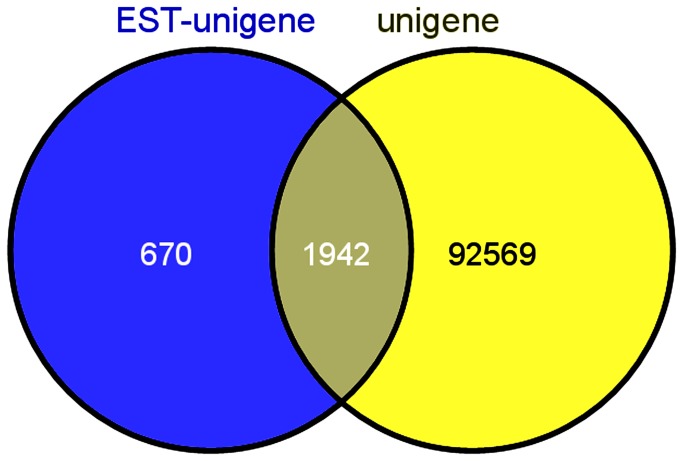
Venn chart for comparisons between assembled transcriptome unigenes and assembled EST-unigenes.

To assess the abundance and coverage of the transcriptome data, we matched the assembled unigenes against the known EST library on Genbank. The 13,985 ESTs downloaded from NCBI were clustered and assembled, and 2,612 assembled EST-unigenes with mean length of 783 bp were generated. Comparisons between transcriptome unigenes and EST-unigenes were performed using BLASTn algorithm. Results were shown in Figure **3** as a Venn chart. 74.3% (1,942 out of 2,612) of the EST-unigenes can be matched in the transcriptome unigenes library, whereas only 2.05% (1,942 out of 94,511) of the transcriptome unigene sequences can be found in the ESTs library.

### Annotation of Unigenes

After ruling out short-length and low-quality sequences, 94,511 unigenes were selected and subjected to annotation analysis by matching sequences against Nr and Swiss-prot databases using BLASTx searching with an E value 1.0×10^-5^ and all annotated unigenes were used to determine the COG term, GO term and KEGG pathway (Table **3** and Figure **S1, S2, S3**). 13,212 unigenes (13.98% of the total) can be matched in Nr database, and 11,711 (12.39% of the total) matched in Swissprot. For main species distribution matched against Nr database, 11.25% of the matched unigenes showed similarities with *Daphnia pulex*, followed by *Tribolium castaneum* (6.90%), *Pediculus humanuscorporis* (5.41%), *Branchiostoma floridae* (3.98%), *Paramecium tetraurelia* (3.58%), *Strongylocentrotus purpuratus* (3.42%), *Nasonia vitripennis* (3.17%), *Ixodes scapularis* (2.90%), *Megachile rotundata* (2.84%), *Acyrthosiphon pisum* (2.66%), *Camponotus floridanus* (2.11%), *Harpegnathos saltator* (2.07%), *Danaus plexippus* (1.98%), *Pediculus humanus* (1.57%), *Tetrahymena thermophila* (1.36%), *Aedes aegypti* (1.36%), and others (43.45%).

**Table 3:** Summary statistics of *Portunus trituberculatus* transcriptome blast assignment.

**Unigenes**	**Nrannotations**	**Swiss-protannotations**	**ESTscan prediction**	**COGhits**	**GOmapped**	**KEGG hits**
**94,511**	13,211	11,711	39,625	11,528	23,914	5,419

### Differentially Expressed Genes

**Figure 4:**
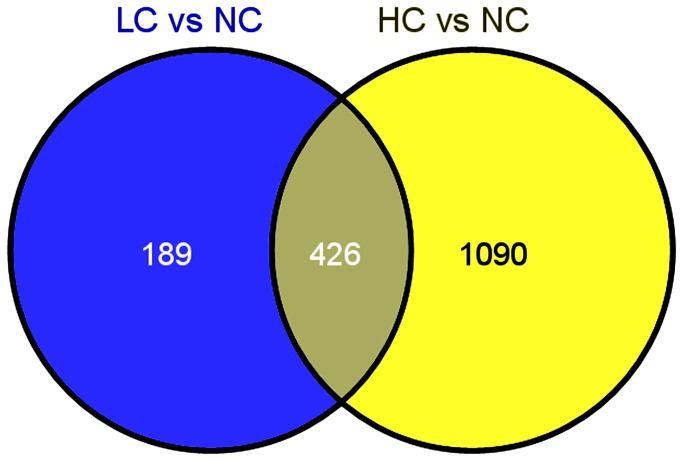
Differentially expressed genes that are unique or shared among LC vs NC and HC vs NC.

Comparison of gene expression showed that a total of 1,705 unigenes were differentially expressed after two different salt challenges (qvalue<0.005 & |log2 (foldchange)|>1), including 615 (158 up and 457 down) differentially expressed unigenes between NC and LC (Table **S2**), and 1516 (895 up and 621 down) between NC and HC (Table **S3**). Moreover, 426 unigenes were significantly differentially expressed in both NC Vs. LC and NC Vs. HC (Figure **4**). To validate our Illumina sequencing results, tweleve differentially regulated genes were selected for quantitative real time-PCR (qRT-PCR) analysis, of which ten genes agrees well with the illumina sequencing analysis.The other two, including sodium-potassium-chloride cotransporter and V-type proton ATPase subunit F, do not match the Illumian sequencing analysis perfectly, however, it shows similar trends in up- or down-regulation of genes analysised by Illumina sequencing (table **4**). The results suggesting that the majority of the salinity responsive genes found from the Illumina sequencing data analysis are authentic. 

**Table 4:** Gene expression levels of 12 selected genes for the comparison of controland salinity-challenged groups (5% or 45%)according to Illumina sequence and qPCR analysis.

**Gene**	**ID**	**qPCR**			**Illumina sequence**	
		LC vs. NC	HC vs. NC		LC vs. NC	HC vs. NC
**glutamine synthetase**	comp33831_c0	0.81	0.20*		0.73	0.16*
**glutamate dehydrogenase**	comp40590_c0	1.10	0.31*		1.20	0.36*
**Sodium / potassium ATPase beta chain**	comp53011_c0	0.11*	0.27*		0.13*	0.25*
**aquaporin**	comp54248_c0	0.32*	0.10*		0.23*	0.01*
**heat shock protein 70**	comp54992_c0	0.53	4.26*		0.64	2.35*
**carbonic anhydrase**	comp55558_c0	7.36*	0.54*		4.73*	0.44*
**V-type proton ATPase subunit B**	comp62089_c0	1.11	0.39*		0.59	0.46*
**sodium-potassium-chloride cotransporter**	comp70314_c0	4.13*	0.31*		1.03	0.26*
**chitinase**	comp71131_c0	0.34*	0.16*		0.23*	0.04*
**V-type proton ATPase subunit F**	comp74516_c3	3.46*	5.39*		1.45	2.13*
**Chloride channel protein**	comp76355_c5	6.71*	1.63		2.80*	0.85
**sodium/hydrogen exchanger**	comp76376_c2	9.10*	1.35*		2.56*	1.81

Asterisk (*) marks the significant difference betweensalinity-challenged group and control group (P < 0.05).

**Figure 5:**
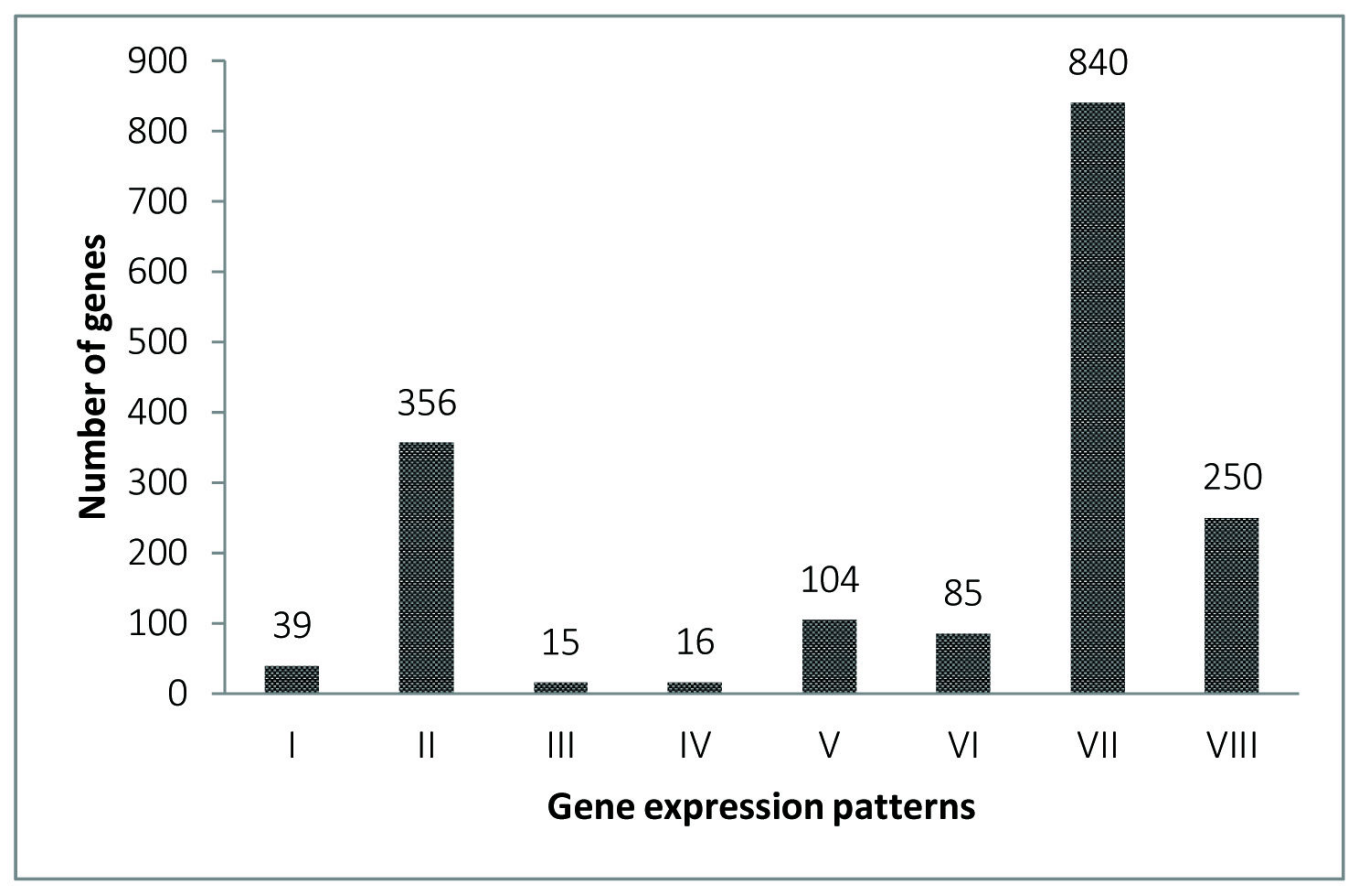
Eight expression patterns ofthe differentially expressed genes. Cluster I, genes highly up-regulated in low and high salinity conditions; Cluster II, genes down-regulated in low and high salinity conditions;Cluster III, genes highly up-regulated in low salinity and down-regulated in high salinity challenge; Cluster IV, genes highly up-regulated in high salinity and down-regulated in low salinity challenge; Cluster V, genes highly up-regulatedonly in low salinity challenge; Cluster VI, genes down-regulation only in low salinity challenge; Cluster VII, genes highly up-regulatedonly in high salinity challenge; Cluster VIII, genes down-regulatedonly in high salinity challenge.

The differentially expressed genes were further categorized into eight patterns based on expression profiles via hierarchical cluster analysis (Figure **5**): genes highly up-regulated in low and high salinity conditions (Cluster I, 39 genes), only in low salinity challenge (Cluster V, 104 genes) and only in high salinity conditions (Cluster VII, 840 genes). These categories are in contrast with three clusters that showed different kinetics of down-regulation in the two salinity conditions (Cluster II, 356 genes), only in low salinity challenge (Cluster VI, 85 genes), and only in high-salinity environment (Cluster VIII, 250 genes). Moreover, Cluster III (15 genes) showed the genes highly up-regulated in low salinity and down-regulated in high salinity challenge, while Cluster IV (16 genes) exhibited the genes highly up-regulated in high salinity and down-regulated in low salinity challenge.

### GO enrichment analysis

**Figure 6:**
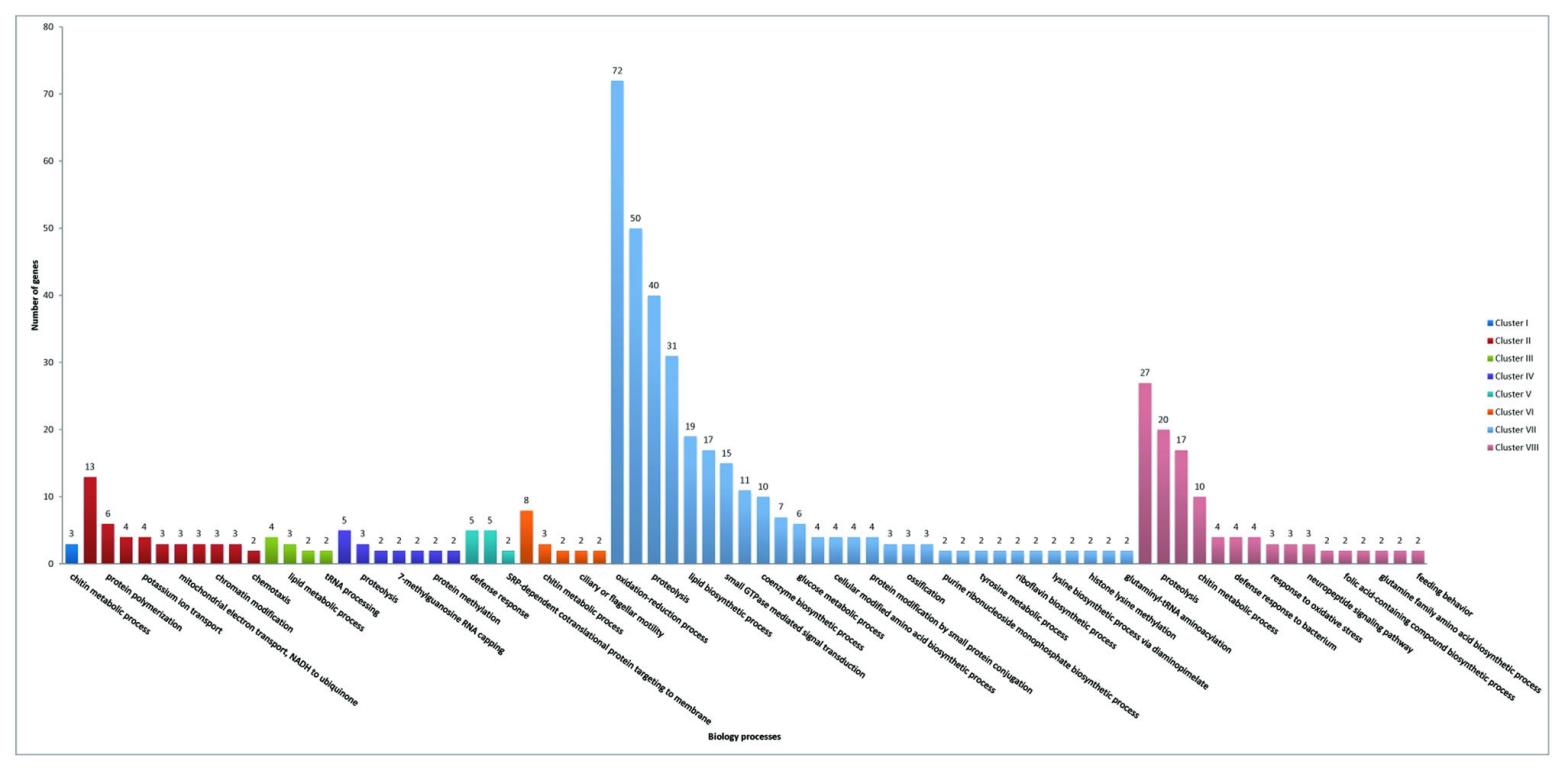
GO enrichment analysis of the differentially expressed genes. Cluster I, genes highly up-regulated in low and high salinity conditions; Cluster II, genes down-regulated in low and high salinity conditions;Cluster III, genes highly up-regulated in low salinity and down-regulated in high salinity challenge; Cluster IV, genes highly up-regulated in high salinity and down-regulated in low salinity challenge; Cluster V, genes highly up-regulatedonly in low salinity challenge; Cluster VI, genes down-regulation only in low salinity challenge; Cluster VII, genes highly up-regulatedonly in high salinity challenge; Cluster VIII, genes down-regulatedonly in high salinity challenge.

Biological process of the differentially expressed genes of the eight patterns were further analyzed according to GO functional enrichment analysis (Figure **6**, Table **5** and Table **S4**). GO functional enrichment analysis results showed cluster I were involved in chitin metabolic process (GO:0006030, 3 unigenes). Cluster II were involved in ten biological processes at a significant level. The mainly processes and involved unigene numbers are chitin metabolic process (GO:0006030, 13 unigenes), protein polymerization (GO:0051258, 6 unigenes), response to oxidative stress (GO:0006979, 4 unigenes), potassium ion transport (GO:0006813, 4 unigenes). Cluster III were involved in four biological processes which mainly include oxidation-reduction process (GO:0055114, 4 unigenes), lipid metabolic process (GO:0006629, 3 unigenes). Cluster IV were involved in five biological processes which mainly include oxidation-reduction process (GO:0055114, 5 unigenes), proteolysis (GO:0006508, 3 unigenes). Cluster V were involved in three biological processes which mainly include defense response (GO:0006952, 5 unigenes), anion transport (GO:0006820, 5 unigenes). Cluster VI were involved in five biological processes which mainly include proteolysis (GO:0006508, 8 unigenes), chitin metabolic process (GO:0006030, 3 unigenes). Additionally, cluster VII and cluster VIII were involved in twenty-nine and sixteen biological processes at a significant level respectively. The mainly processes, involved unigene numbers and cluster numbers are oxidation-reduction process (GO:0055114, 72 unigenes, cluster VII), cellular protein modification process (GO:0006464, 50 unigenes, cluster VII), proteolysis (GO:0006508, 40 unigenes, cluster VII), phosphorylation (GO:0016310, 31 unigenes, cluster VII), oxidation-reduction process (GO:0055114, 27 unigenes, cluster VIII), proteolysis (GO:0006508, 20 unigenes, cluster VIII), carbohydrate metabolic process (GO:0005975, 17 unigenes, cluster VIII), chitin metabolic process (GO:0006030, 10 unigenes, cluster VIII).

**Table 5:** GO enrichment analysis of the differentially expressed genes.

**Cluster**	**GOID**	**Term**	**Level of GO**	**Number of unigenes**	***P*-value**
**I**	GO:0006030	chitin metabolic process	6	3	0.001204934
**II**	GO:0006030	chitin metabolic process	6	13	1.60E-08
**II**	GO:0051258	protein polymerization	7	6	0.000898448
**II**	GO:0006813	potassium ion transport	8	4	0.032189626
**II**	GO:0006979	response to oxidative stress	3	4	0.000251565
**II**	GO:0006120	mitochondrial electron transport, NADH to ubiquinone	9	3	0.037277062
**II**	GO:0006875	cellular metal ion homeostasis	8	3	0.041235929
**II**	GO:0009755	hormone-mediated signaling pathway	7	3	0.037277062
**II**	GO:0016568	chromatin modification	6	3	0.043988317
**II**	GO:0071941	nitrogen cycle metabolic process	3	3	0.005597731
**II**	GO:0006935	chemotaxis	4	2	0.048209006
**III**	GO:0055114	oxidation-reduction process	3	4	0.017862676
**III**	GO:0006629	lipid metabolic process	3	3	0.035596858
**III**	GO:0006546	glycine catabolic process	10	2	9.13E-06
**III**	GO:0008033	tRNA processing	9	2	0.002337968
**IV**	GO:0055114	oxidation-reduction process	3	5	0.043166178
**IV**	GO:0006508	proteolysis	5	3	0.031323303
**IV**	GO:0002098	tRNA wobble uridine modification	12	2	0.000102853
**IV**	GO:0000154	rRNA modification	10	2	0.000190739
**IV**	GO:0009452	7-methylguanosine RNA capping	9	2	0.000190739
**IV**	GO:0001510	RNA methylation	8	2	0.000395991
**IV**	GO:0006479	protein methylation	8	2	0.000770984
**V**	GO:0006820	anion transport	6	5	5.52E-05
**V**	GO:0006952	defense response	3	5	0.034822636
**V**	GO:0006614	SRP-dependent cotranslational protein targeting to membrane	10	2	0.008743629
**VI**	GO:0006508	proteolysis	5	8	0.003397869
**VI**	GO:0006030	chitin metabolic process	6	3	0.01432079
**VI**	GO:0006888	ER to Golgi vesicle-mediated transport	7	2	0.037838935
**VI**	GO:0009878	nodule morphogenesis	6	2	0.021455881
**VI**	GO:0001539	ciliary or flagellar motility	5	2	0.001800326
**VII**	GO:0055114	oxidation-reduction process	3	72	0.000952872
**VII**	GO:0006464	cellular protein modification process	6	50	0.000230686
**VII**	GO:0006508	proteolysis	5	40	0.000397024
**VII**	GO:0016310	phosphorylation	5	31	0.001296757
**VII**	GO:0008610	lipid biosynthetic process	4	19	0.003426418
**VII**	GO:0009966	regulation of signal transduction	5	17	0.041422297
**VII**	GO:0007264	small GTPase mediated signal transduction	6	15	0.000113889
**VII**	GO:0006030	chitin metabolic process	6	11	0.006871104
**VII**	GO:0009108	coenzyme biosynthetic process	5	10	0.001612753
**VII**	GO:0006888	ER to Golgi vesicle-mediated transport	7	7	0.021852563
**VII**	GO:0006006	glucose metabolic process	7	6	0.038801546
**VII**	GO:0042398	cellular modified amino acid biosynthetic process	9	4	0.04770803
**VII**	GO:0035023	regulation of Rho protein signal transduction	8	4	0.008616499
**VII**	GO:0032446	protein modification by small protein conjugation	8	4	0.039101467
**VII**	GO:0007339	binding of sperm to zona pellucida	7	4	0.000990193
**VII**	GO:0006099	tricarboxylic acid cycle	7	3	0.047820393
**VII**	GO:0006000	fructose metabolic process	7	3	0.007238233
**VII**	GO:0001503	ossification	3	3	0.043900004
**VII**	GO:0009089	lysine biosynthetic process via diaminopimelate	11	2	0.04770803
**VII**	GO:0015813	L-glutamate transport	11	2	0.018737173
**VII**	GO:0006425	glutaminyl-tRNA aminoacylation	11	2	0.018737173
**VII**	GO:0009168	purine ribonucleoside monophosphate biosynthetic process	10	2	0.041097287
**VII**	GO:0034968	histone lysine methylation	10	2	0.03486768
**VII**	GO:0046058	cAMP metabolic process	10	2	0.02365927
**VII**	GO:0006570	tyrosine metabolic process	9	2	0.014309718
**VII**	GO:0006541	glutamine metabolic process	9	2	0.04770803
**VII**	GO:0006784	heme a biosynthetic process	8	2	0.010408479
**VII**	GO:0009231	riboflavin biosynthetic process	7	2	0.029045762
**VII**	GO:0030534	adult behavior	4	2	0.03486768
**VIII**	GO:0055114	oxidation-reduction process	3	27	0.001223294
**VIII**	GO:0006508	proteolysis	5	20	1.19E-05
**VIII**	GO:0005975	carbohydrate metabolic process	3	17	6.23E-05
**VIII**	GO:0006030	chitin metabolic process	6	10	2.79E-06
**VIII**	GO:0006544	glycine metabolic process	9	4	0.000286422
**VIII**	GO:0006631	fatty acid metabolic process	8	4	0.013582145
**VIII**	GO:0042742	defense response to bacterium	5	4	0.020716006
**VIII**	GO:0006825	copper ion transport	9	3	0.002850419
**VIII**	GO:0007218	neuropeptide signaling pathway	7	3	0.042788588
**VIII**	GO:0006979	response to oxidative stress	3	3	0.002600038
**VIII**	GO:0006334	nucleosome assembly	10	2	0.01916756
**VIII**	GO:0009396	folic acid-containing compound biosynthetic process	10	2	0.01916756
**VIII**	GO:0006563	L-serine metabolic process	9	2	0.00077586
**VIII**	GO:0009084	glutamine family amino acid biosynthetic process	9	2	0.005774778
**VIII**	GO:0008272	sulfate transport	8	2	0.011002118
**VIII**	GO:0007631	feeding behavior	3	2	0.020714011

## Discussion

In recent years, the next generation sequencing methods have also been applied to analyze transcriptomes of crustaceans using Illumina sequencing. Comparing with the traditional methods, the next-generation high-throughput DNA sequencing techniques provide more ideal methods for transcriptome analyses with high efficiency, low cost and high data output. The former studies on *Portunus trituberculatus* transcriptome were performed using traditional cDNA library and Sanger sequencing methods with RNA from many organs such as gill, eyestalk, blood and hepatopancreas [28-31], however, it remains insufficient for the comprehensive understanding of *Portunus trituberculatus* transcriptome. In our study, we generated 141,339 contigs of *Portunus trituberculatus* transcriptome based on the next generation sequencing techniques. Further assembly analysis showed that all contigs contributed to 94,511 unigenes, largely enriching the transcriptome data of *Portunus trituberculatus* and prompting the genome studies of crustaceans.

We compared our transcriptome data with *Portunus trituberculatus* EST sequences obtained from NCBI and showed that more than half of the EST sequences (74.3%) can be matched in the transcriptome data, whereas only 2.05% of the transcriptome unigene sequences can be found in the ESTs library. It suggests the transcriptome data provide abundant information besides the now available ESTs sequences. After a homology search in the non-redundant protein database at NCBI, a total of 13,212 unigenes, which took up a proportion of 13.98% in all the unigenes, showed significant BlastX hits of known protein sequences. The distribution of significant BlastX hits over different organisms was also analyzed. Due to the lack of genomic information in *Portunus trituberculatus*, the majority of the assembled sequences (11.25%) matched genes from microcrustacean arthropod *Daphnia pulex* which have full genomic information.

Comparison of gene expression among the different treatment groups in the current experiment is helpful for identification of candidate genes underlying response to salinity stress in *Portunus trituberculatus*. In this study, we detected a total of 1,705 unigenes were differentially expressed between the comparison of LC vs NC and HC vs NC (qvalue<0.005 & |log2 (foldchange)|>1), which significantly more abundant than the data of previous study, in which only 417 differentially expressed genes were found after two different salt challenges (10% and 40%) via cDNA microarray technology [5]. The results prove that the next generation sequencing methods should be more powerful than cDNA microarray technology in expression analysis because it can provide high data output and recognize new unigenes or unique isoforms present in transcriptome [32].

Many fewer differentially expressed unigenes were found in the LC Vs. NC (615) compared with that of HC Vs. NC (1,516). To further unravel the significantly altered biological processes upon salinity stress, the up- and down-regulated genes categorized into eight patterns based on expression profiles were subjected to the GO term enrichment analysis. The results revealed that a total of 454 unigenes were enrichmented in 63 processes, among which, the differentially expressed unigenes in low salinity stress were enrichmented in 25 processes (6 processes were induced, 17 processes were suppressed and 2 processes were were affected). The differentially expressed unigenes in high salinity stress were enrichmented in 58 processes (31 processes were induced, 24 processes were suppressed and 3 processes were were affected). Further analysis found, 5 and 38 processes were only enrichmented in low salinity stress or high salinity stress respectively, another 20 processes were enrichmented in both stress. In conclusion, our analysis uncovered: 1, more genes and processes were involved in high salinity stress adaptation than low salinity stress adaptation. 2, the processed involved in low salinity stress were mainly supressed, and the processed involved in high salinity stress were mainly induced. 3, hypo-osmoregulation and hyper-osmoregulation mechanisms share some processes but also has many differences.

Past research has revealed two major strategies, i.e. the “limiting process” and the “compensatory process”, that are adopted by crustaceans for osmoregulation and both are predominately accomplished by the gills [33,34]. A “limiting process”is a strategy whereby the maintenance of hemolymph osmolality/ions is accomplished by adjusting the permeability of the boundary structures (e.g. gill mem-branes), which can be a highly effective method to reduce ion diffusion and water inux rather than solely relying on the more energetically demanding mechanisms of ion transport. It has been suggested that the mechanisms during long term salinity exposure is often the result of gill membrane fatty acid compositional changes [35]. In the case, generally membranes containing higher proportions of fatty acids with high unsaturation indexes (i.e. higher double bonds). In this research, we found at least five up-regulation unigenes belong to fatty acid biosynthetic process (GO:0006631) in response to salinity stress (Table **S4**) which suggested the strategies of “limiting process” maybe play an important role in osmoregulation in *Portunus trituberculatus*.

A “compensatory process”, on the other hand, is a strategy whereby the maintenance of hemolymph osmolality/ions is accomplished via the active movement of solutes into or out of the hemolymph to counter balance their passive diffusion [4,33]. When the environmental osmolality is higher than that of the hemolymph, it is termed “hyper-osmotic” and crustaceans must compensate for ion inﬂux via hypo-osmoregulation; for the opposite, when the environmental osmolality is lower than that of the hemolymph, it is termed “hypo-osmotic” and crustaceans must compensate for ion loss from their hemolymph via hyper-osmoregulation [34]. *Portunus trituberculatus* belongs to *weak* hyperosmoregulators [36] which employs the apical Na^+,^ K^+^, 2Cl(^-^) cotransporter driven by the inwardly directed Na^+^ gradient, supplemented by apical K^+^ channels, that recycle K^+^ and hyperpolarize the apical membrane, creating a negative cell potential that drives Cl^-^ efﬂux through basal Cl^-^ channels. Na^+^ uptake may be augmented through the apical sodium/hydrogen exchanger. The resulting outside-positive transepithelial potential drives substantial inward paracellular Na^+^ movement across this leaky epithelium [36,37]. In our study, ion transport-related genes including chloride channel protein, sodium/hydrogen exchanger, sodium/glucose cotransporter, and carbonic anhydrase were up-regulated in response to low salinity stress. In addition, we found that five signifantly up-regulated expressed unigenes were enrichmented in anion transport process (GO:0006820). The differential expression of this key ion transport genes potentially supports this models of decapod Crustacea hyper-osmoregulation reported previously [36,37]. 

On the contrary, in hypo-osmoregulation, passive NaCl influx is compensated by active NaCl secretion, however, the mechanisms of active NaCl secretion in the Crustacea are not known[36]. In this studys, we also found ion transport-related genes in high salinity stress, but most of them down-regulated expression which were different from low salinity stress. These down-regulated genes contained Na^+^, K^+^, 2Cl(^-^) cotransporter, carbonic anhydrase, calcium-activated chloride channel regulator 1, and anion exchange protein etc. Based on previous researches [36,37] and our results, we speculated that: in hypo-osmoregulation, for maintaining hemolymph osmotic balance, *Portunus trituberculatus* initiatively down-reglated the expression of some important ion transport-related genes to prevent high concentration of ions of the external environment penetrate into the body. However, more detailed studies are required to disclose the mechanisms of hypo-osmoregulation in Crustacea.

The aquaporins were considered as important salt responsive effectors [38]. The functions of AQP have been studied in mammals [39] and fish [40] which suggested that epithelial water flux occurs, at least in part, through transcellular pathways formed via aquaporins (AQPs), however, the functions of aquaporins in osmoregulation was little known in crustacea. Previous researches suggested that during acclimation to a hyperosmotic environment, increasing renal AQPs expression may play a role in enhancing water reabsorption by the kidney [41] and translational gene knockdown of AQPs protein reduced water influx in fish [42]. Additionally, the water channel activity of AQPs can be modulated through phosphorylation and dephosphorylation [43-45]. In addition to maintaining ionic homeostasis under salt stress conditions, *Portunus trituberculatus* also need establish water homeostasis via aquaporins (AQPs). Our data showed that the mRNA expression level of AQPs decreased under hypo-osmotic and hyper-osmotic stress conditions. Similar results were found in many previous studies under long-term saltstress [38,46]. These results may indicated that long-term stress induced a reduction of AQPs activities via transcriptional level controls and post-translational modiﬁcations to protect against water currents as well as gill cell swelling and shrinkage. However, AQPs activities whether affected by phosphorylation in *Portunus trituberculatus* should be further verified.

It has been reported that Na^+^,K^+^-ATPase is the driving force in establishing an ion gradient across the epithelial cell membrane in marine crabs [47]. In this study, Na^+^,K^+^-ATPase β-subunit showed significant down-regulation in high and low salinity conditions, which suggested that Na^+^,K^+^-ATPase might play a very important role in salinity adaptation. Previous studies have shown that Na^+^,K^+^-ATPase β-subunit showed significant up-regulation during low salinity challenge and significant down-regulation during high salinity challenges [5], which was different from our data. This may be due to different salinity challenged in the two studies. Although lots of literature showed that gene expression levels of Na^+^,K^+^-ATPase α subunit were highly up-regulated during salinity stress [48-50], significant up-/down-regulation of Na^+^,K^+^-ATPase α subunit was not detected in our transcriptome data, and similar results were also found in Xu’s studies [5]. None of the significant gene expression changes of Na^+^,K^+^-ATPase α subunit during salinity challenges do not negate its role for osmoregulation in swimming crab, and more future researches should be done toward Na^+^,K^+^-ATPase α subunit.

Previous studies have shown that the gill apical V-H^+^-ATPase in freshwater crabs was shown to be involved in ion regulation, and marine ones generally showed a cytoplasmic V-H^+^-ATPase distribution, which did not participate in osmoregulation [51]. Similar suggestions have already been proposed in studies of the gills of *Eriocheir sinensis* and *Uca tangeri* [52-54]. *Portunus trituberculatus* belong to typical euryhaline marine crab species, however, it is interesting that four V-H+-ATPase subunits were showed significantly different expresstion in high salinity stress, which suggested V-H^+^-ATPase might play a very important role during hypo-osmoregulation. Subcellular localization and function of V-H^+^-ATPase deserve further study in *Portunus trituberculatus.*

The present investigation suggests that the degradation of hemolymph or muscle protein to FAAs or de novo synthesis of free amino acids (FAA) may serve as a mechanism to compensate or alleviate the effects of ion influx under salinity stress [55], and that glycine, proline and alanine widely function as osmoeffectors in a number of crustacean species [56-59]. It must be noted, however, such a response is not universal to all crustaceans [60]. In this study, many differentially expressed unigenes were enrichmented in amino acid metabolism and synthesis processes. Among which, glycine catabolic process (GO:0006546) was induced in low salinity stress, and in high salinity stress, glycine metabolic process (GO:0006544) and L-serine metabolic process (GO:0006563) were suppressed, but lysine biosynthetic process (GO:0009089) was induced. Interestingly, we aslo found many differentially expressed unigenes enriched in proteolysis process (GO:0006508), which down-regulated (11 genes) in low salinity stress and mainly up-regulated (43 up-regulated genes and 20 down-regulated genes) in high salinity stress. This results uncovered that, in high salinity stress, to balance the osmotic pressure inside and outside the body, *Portunus trituberculatus* increase levels of FAAs in vivo via the way of increasing protein degradation, decreaseing amino acid catabolism, and increasing synthesis of certain non-essential amino acids, and in low salinity stress, *Portunus trituberculatus* takes the opposite approach to hypertonic regulation.

In addition to the known processes associated with osmoregulation in Crustacea., there were some other processes enriched which were little known in osmoregulation. The top three processes based on the number of unigenes were oxidation-reduction process (GO:0055114), cellular protein modification process (GO:0006464) and phosphorylation (GO:0016310), and the top three processes based on the enrichment level (*p*-value, excluding the processes of less than three unigenes) were chitin metabolic process (GO:0006030), carbohydrate metabolic process (GO:0005975) and small GTPase mediated signal transduction (GO:0007264).

The oxidation-reduction process is essential in maintaining cellular homeostasis. Under physiologic conditions, cells maintain oxidation-reduction balance through generation and elimination of reactive oxygen/nitrogen species (ROS/RNS) [61]. Normally, the oxidation-reduction process homeostasis ensures that the cells respond properly to endogenous and exogenous stimuli. However, when the oxidation-reduction process homeostasis is disturbed, oxidative stress may lead to aberrant cell death and contribute to disease development [61]. Both exogenous and endogenous sources contribute to the formation of intracellular ROS/RNS. This reseach suggested that exposure to salinity stress, especially the high salinity stress has been shown to active the oxidation-reduction process in *Portunus trituberculatus* in order to balance the level of ROS/RNS. Of course, there was another possibility that the oxidation-reduction process in *Portunus trituberculatus* has been disturbed in this high salinity stress. Both cellular protein modification and phosphorylation process belong to the scope of post-translational modifications. 

Posttranslational modifications modulate the activity of most eukaryote proteins [62] which. manifest as chemical modifications that occur on amino acid side chains in a site-specific manner. They can temporarily or permanently change the fate of the protein by enhancing the functionality and/or stability of the target protein through the recruitment of auxiliary factors, change the proteins' cellular localisation or signal the most terminal fate, proteasomal degradation [63]. Our data showed that many gene were enrichmented in cellular protein modification (50 unigenes ) and phosphorylation process (31 unigenes ), suggested that posttranslational modifications play an important role in osmoregulation of *Portunus trituberculatus*, and further research is worthwhile.

Many differentially expressed unigenes (38) enriched in chitin metabolic process with the maximum enrichment level (*p*-value: 1.60E-08) were aslo found in our data, which suggested chitin metabolic process may be involved in osmoregulation or affected by salinity stress. Further analysis found there were 4 chitinase genes. In crustaceans, during the molting cycle, chitinase dissolves chitin in the old exoskeleton into more soluble forms, which can then be partially reabsorbed into the body and used to synthesize the new exoskeleton [64]. Therefore, the chitinase are indispensable for crustaceans. By now, many genes encoding chitinases have been isolated and characterized in crustaceans [64-67] due to their importance in growth, development and immunity. However, chitinase with function of osmoregulation has not been reported in crustacea. More detailed research can be done according to the connections between the salinity changes and chitin metabolic process in the future.

Small GTP-binding genes play a crucial regulatory role in a number of cellular processes in both plants and ani-mals, such as vesicle-mediated intracellular trafﬁcking, signal transduction, cytoskeletal organization, and cell division [68]. Differential expression of small GTP-binding proteins has been previously reported in various abiotic stresses in plant [68], animal [69], and bacteria [70], however, the functions of Small GTP-binding genes was little known in crustacea. 15 unigenes were enriched in GTPase mediated signal transduction process with a relatively low *p*-value implied that GTPase mediated signal transduction process plays an important role in *Portunus trituberculatus* salinity adaptation. In addition, 17 up-regulated unigenes were enriched in carbohydrate metabolic process in high salinity stress suggested that hypo-osmoregulation of *Portunus trituberculatus* depends on energy consumption. This result have also been reported in Xu’s studies [5] and further experiments are needed to verify the hypothesis.

It should be noted that, the processes of response to oxidative stress (GO:0006979), defense response (GO:0006952), response to oxidative stress (GO:0006979) and defense response to bacterium (GO:0042742) were enriched in salinity stess, and lots of stress-related and immunity-related genes such as HSP, cathepsin, lectin, serine protease, and peroxiredoxin show significantly up-regulated or down-regulation expression responding to low or high salinity stress, which suggested that salinity dilution or elevation appeared to invoke a classic ‘‘stress response’’ and “immunity response” at the transcriptional level in *Portunus trituberculatus*. This results similar to Xu et al.(2011)’s results [5] and different from Towle et al.(2011)’s results [71], which might reflect that *Portunus trituberculatus* possesses lower salinity adaptability than *C. maenas*, and it also suggested that the two crab species might have different signal pathways against the salinity ‘‘stress’’[5].

It is noteworthy that the present study identiﬁed a large number of transcripts (526 unigenes) signiﬁcantly upregulated or downregulate during salinity stress for which no annotation was readily available. This new genes provide a wealth of reference data to further research the mechanism of osmoregulation in Crustacea.

## Conclusions

In general, our work represents the first report of the utilization of the next generation sequencing techniques for the study of osmoregulation in *Portunus trituberculatus*. Through more than 18.16G clean bases obtained, we established transcriptome data set for *Portunus trituberculatus* subjected to salinity stress, and the data we generated could enrich on genomic resources of this non-model organism. Based on the comparison of gene expression, a substantial number of genes were found to be modified by salinity stress which demonstrated the complexity of salinity adaptation mechanism in the crab. Our results revealed a few important salinity acclimation pathways via enrichment analysis, which may be helpful in understanding the molecular basis of osmoregulation in *Portunus trituberculatus*.

## Supporting Information

Table S1 **Primer sequences and product size of target and reference genes used for quantitative RT-PCR.**
(XLSX)

Table S2 **List of differently expressed genes from Portunus trituberculatus in response to low salinity challenged (LC) versus non-challenged (NC).**
(XLSX)

Table S3 **List of differently expressed genes from Portunus trituberculatus in response to high salinity challenged (HC) versus non-challenged (NC).**
(XLSX)

Figure S1 **COG Classification of the unigenes.** Possible functions of 11528 unigenes were classified and subdivided into 26 COG categories.
(JPG)

Figure S2 **GO classification of all unigenes.** Most unigenes canbe divided into three major categories, including biological process, cellular component, and molecular function.
(JPG)

Figure S3 **KEGG Classification of the unigenes.** 5419 unigenes were assigned into 31 KEGG pathways.A, Cellular Processes; B, Enviromental Information Processing; C, Genetic Information Processing; D, Metabolism; E, Organismal Systems.
(PNG)

## References

[1] Dai A, Yang S, Song Y (1986) Marine crabs in China Sea. Marine Publishing Company, Beijing.

[2] Yu C, Song H, Yao G (2003) Geographical distribution and faunal analysis of crab resources in the East China Sea. J Zhejiang Ocean Univ 22: 108-113

[3] Liu L, Li J, Liu P, Zhao FZ, Gao BQ et al. (2012) A genetic linkage map of swimming crab (Portunus trituberculatus) based on SSR and AFLP markers. Aquaculture 344: 66-81.

[4] Romano N, Zeng CS (2012) Osmoregulation in decapod crustaceans: implications to aquaculture productivity, methods for potential improvement and interactions with elevated ammonia exposure. Aquaculture 334: 12-23.

[5] Xu QH, Liu Y (2011) Gene expression profiles of the swimming crab Portunus trituberculatus exposed to salinity stress. Marine Biology 158: 2161-2172. doi:10.1007/s00227-011-1721-8.

[6] Charmantier G, Charmantier-Daures M, Towle D (2008) Osmotic and ionic regulation in aquatic arthropods.

[7] Fu L, Jintian H, Yebing Y, Wenping Y (2010) AFFECTS OF SALINITY ON GROWTH, MUSCLE COMPOSITION,AND PROTEASE ACTIVITY OF PORTUNUS TRITUBERCULATUS. Transactions of Oceanology and Limnology: 137-142

[8] Ping-ping Z, Chun-lin W, wei SW, Dan-hua W (2010) Effect of Salinity Stress on Serum Non-specific Immune Factors in Swimming Crab; Portunus trituberculatus. Fisheries Science: 634-638.

[9] Shan J, Qiang-hua X (2011) Influence of salinity stress on the activity of gill Na+/K+-ATPase in swimming crab( Portunus trituberculatus). Journal Fisheries of China: 1475-1480.

[10] Yan-ming S, Bao-quan G, Ping L, Xian-yun R, Yang L, et al. (2012) The tolerance to and optimal salinity for growth in swimming crab Portunus trituberculatus “Huangxuan No. 1”. JOURNAL OF DALIAN OCEAN UNIVERSITY : 398-401

[11] Yun-liang L, Fang W, Qin-feng G, Shuang-lin D (2012) Effects of salinity on the respiratory metabolism of pre-and post-maturity swimming crab( Portunus trituberculatus). Journal Fisheries of China: 1392-1399.

[12] Yunliang L, Fang W, Zhuoying Z, Shuanglin D, Shen M (2012) Effects of salinity on growth, molt and energy utilization of juvenileswimming crab Portunus trituberculatus. Journal Fishery Sciences of China: 237-245.

[13] Wang Z, Gerstein M, Snyder M (2009) RNA-Seq: a revolutionary tool for transcriptomics. Nat Rev Genet 10: 57-63. doi:10.1038/nrg2484. PubMed: 19015660. 19015660 PMC2949280

[14] Ramayo-Caldas Y, Mach N, Esteve-Codina A, Corominas J, Castelló A et al. (2012) Liver transcriptome profile in pigs with extreme phenotypes of intramuscular fatty acid composition. BMC Genomics 13: 547-. PubMed: 23051667. 23051667 10.1186/1471-2164-13-547 PMC3478172

[15] Sánchez CC, Weber GM, Gao G, Cleveland BM, Yao J et al. (2011) Generation of a reference transcriptome for evaluating rainbow trout responses to various stressors. BMC Genomics 12: 626-. PubMed: 22188770. 22188770 10.1186/1471-2164-12-626 PMC3305546

[16] Wang XW, Luan JB, Li JM, Su YL, Xia J et al. (2011) Transcriptome analysis and comparison reveal divergence between two invasive whitefly cryptic species. BMC Genomics 12: 458-. PubMed: 21939539. 21939539 10.1186/1471-2164-12-458 PMC3189941

[17] Zeng D, Chen X, Xie D, Zhao Y, Yang C et al. (2013) Transcriptome Analysis of Pacific White Shrimp (<italic>Litopenaeus vannamei</italic>) Hepatopancreas in Response to Taura Syndrome Virus (TSV) Experimental. Infection - PLOS ONE 8: e57515. doi:10.1371/journal.pone.0057515. 23469011 PMC3585375

[18] Li S, Zhang X, Sun Z, Li F, Xiang J (2013) Transcriptome Analysis on Chinese Shrimp <italic>Fenneropenaeus chinensis</italic> during WSSV Acute. Infection - PLOS ONE 8: e58627. doi:10.1371/journal.pone.0058627. 23527000 PMC3602427

[19] He L, Jiang H, Cao D, Liu L, Hu S, et al. (2013) Comparative Transcriptome Analysis of the Accessory Sex Gland and Testis from the Chinese Mitten Crab (<italic>Eriocheir sinensis</italic>). PLoS ONE 8: e53915 10.1371/journal.pone.0053915 PMC3547057 23342039

[20] Ma KY, Qiu GF, Feng JB, Li JL (2012) Transcriptome Analysis of the Oriental River Prawn, Macrobrachium nipponense Using 454 Pyrosequencing for Discovery of Genes and Markers. PLOS ONE 7: e39727 PubMed: 22745820. 22745820 10.1371/journal.pone.0039727 PMC3380025

[21] He L, Wang Q, Jin XK, Wang Y, Chen LL et al. (2012) Transcriptome Profiling of Testis during Sexual Maturation Stages in Eriocheir sinensis Using Illumina Sequencing. PLOS ONE 7: e33735 PubMed: 22442720. 22442720 10.1371/journal.pone.0033735 PMC3307765

[22] Marguerat S, Bähler J (2010) RNA-seq: from technology to biology. Cell Mol Life Sci 67: 569-579. doi:10.1007/s00018-009-0180-6. PubMed: 19859660. 19859660 PMC2809939

[23] Grabherr MG, Haas BJ, Yassour M, Levin JZ, Thompson DA et al. (2011) Full-length transcriptome assembly from RNA-Seq data without a reference genome. Nat Biotechnol 29: 644-652. doi:10.1038/nbt.1883. PubMed: 21572440. 21572440 PMC3571712

[24] Altschul SF, Madden TL, Schäffer AA, Zhang J, Zhang Z et al. (1997) Gapped BLAST and PSI-BLAST: a new generation of protein database search programs. Nucleic Acids Res 25: 3389-3402. doi:10.1093/nar/25.17.3389. PubMed: 9254694. 9254694 PMC146917

[25] Storey JD, Tibshirani R (2003) Statistical significance for genomewide studies. Proc Natl Acad Sci U S A 100: 9440-9445. doi:10.1073/pnas.1530509100. PubMed: 12883005. 12883005 PMC170937

[26] de Hoon MJ, Imoto S, Nolan J, Miyano S (2004) Open source clustering software. Bioinformatics 20: 1453-1454. doi:10.1093/bioinformatics/bth078. PubMed: 14871861. 14871861

[27] Young MD, Wakefield MJ, Smyth GK, Oshlack A (2010) Method Gene ontology analysis for RNA-seq: accounting for selection bias. 10.1186/gb-2010-11-2-r14 PMC2872874 20132535

[28] Xu QH, Liu Y, Liu RL (2010) Expressed sequence tags from cDNA library prepared from gills of the swimming crab, Portunus trituberculatus. Journal of Experimental Marine Biology and Ecology 394: 105-115. doi:10.1016/j.jembe.2010.08.002.

[29] Liu Y, Cui ZX, Song CW, Wang SY, Li QQ (2011) Multiple isoforms of immune-related genes from hemocytes and eyestalk cDNA libraries of swimming crab Portunus trituberculatus. Fish Shellfish Immunol 31: 29-42. PubMed: 21362485. 21362485 10.1016/j.fsi.2011.02.016

[30] Wang SY, Cui ZX, Liu Y, Li QQ, Song CW (2012) The first homolog of pacifastin-related precursor in the swimming crab (Portunus trituberculatus): Characterization and potential role in immune response to bacteria and fungi. Fish Shellfish Immunol 32: 331-338. PubMed: 22154999. 22154999 10.1016/j.fsi.2011.11.025

[31] Wang SY, Cui ZX, Liu Y, Li QQ, Song CW (2012) Identification and characterization of a serine protease inhibitor (PtSerpin) in the swimming crab Portunus trituberculatus. Fish Shellfish Immunol 32: 544-550. PubMed: 22245590. 22245590 10.1016/j.fsi.2012.01.002

[32] Nie QH, Sandford EE, Zhang XQ, Nolan LK, Lamont SJ (2012) Deep Sequencing-Based Transcriptome Analysis of Chicken Spleen in Response to Avian Pathogenic Escherichia coli (APEC). Infection - PLOS ONE 7. 10.1371/journal.pone.0041645 PMC3409229 22860004

[33] Péqueux A (1995) Osmotic regulation in crustaceans. Journal of Crustacean Biology 15: 1-60. doi:10.1163/193724095X00578.

[34] Rainbow P, Black W (2001) Effects of changes in salinity on the apparent water permeability of three crab species:< i > Carcinus maenas</i>,< i > Eriocheir sinensis</i> and< i> Necora puber</i>. Journal of Experimental Marine Biology and Ecology 264: 1-13

[35] Morris R, Lockwood A, Dawson M (1982) An effect of acclimation salinity on the fatty acid composition of the gill phospholipids and water flux of the amphipod crustacean< i> Gammarus duebeni</i >. Comparative Biochemistry and Physiology Part A: Physiology 72: 497-503

[36] Freire CA, Onken H, McNamara JC (2008) A structure-function analysis of ion transport in crustacean gills and excretory organs. Comparative Biochemistry and Physiology A:_Molecular and Integrative Physiology 151: 272-304. doi:10.1016/j.cbpa.2007.05.008. 17604200

[37] McNamara JC, Faria SC (2012) Evolution of osmoregulatory patterns and gill ion transport mechanisms in the decapod Crustacea: a review. J Comp Physiol B 182: 997-1014. doi:10.1007/s00360-012-0665-8. PubMed: 22534792. 22534792

[38] Meng J, Zhu Q, Zhang L, Li C, Li L, et al. (2013) Genome and Transcriptome Analyses Provide Insight into the Euryhaline Adaptation Mechanism of <italic>Crassostrea gigas</italic >. PLoS ONE 8: e58563 10.1371/journal.pone.0058563 PMC3595286 23554902

[39] King LS, Kozono D, Agre P (2004) From structure to disease: the evolving tale of aquaporin biology. Nat Rev Mol Cell Biol 5: 687-698. doi:10.1038/nrm1469. PubMed: 15340377. 15340377

[40] Cerdà J, Finn RN (2010) Piscine aquaporins: an overview of recent advances. J Exp Zool A Ecol Genet Physiol 313: 623-650. PubMed: 20717996. 20717996 10.1002/jez.634

[41] Tipsmark CK, Sørensen KJ, Madsen SS (2010) Aquaporin expression dynamics in osmoregulatory tissues of Atlantic salmon during smoltification and seawater acclimation. J Exp Biol 213: 368-379. doi:10.1242/jeb.034785. PubMed: 20086120. 20086120

[42] Kwong RWM, Kumai Y, Perry SF (2013) The Role of Aquaporin and Tight Junction Proteins in the Regulation of Water Movement in Larval Zebrafish (Danio rerio). PLOS ONE 8. 10.1371/journal.pone.0070764 PMC3743848 23967101

[43] Tournaire-Roux C, Sutka M, Javot H, Gout E, Gerbeau P et al. (2003) Cytosolic pH regulates root water transport during anoxic stress through gating of aquaporins. Nature 425: 393-397. doi:10.1038/nature01853. PubMed: 14508488. 14508488

[44] Santoni V, Vinh J, Pflieger D, Sommerer N, Maurel C (2003) A proteomic study reveals novel insights into the diversity of aquaporin forms expressed in the plasma membrane of plant roots. Biochem J 373: 289-296. doi:10.1042/BJ20030159. PubMed: 12678916. 12678916 PMC1223478

[45] Azad AK, Katsuhara M, Sawa Y, Ishikawa T, Shibata H (2008) Characterization of four plasma membrane aquaporins in tulip petals: A putative homolog is regulated by phosphorylation. Plant Cell Physiol 49: 1196-1208. doi:10.1093/pcp/pcn095. PubMed: 18567892. 18567892

[46] Boursiac Y, Chen S, Luu D-T, Sorieul M, van den Dries N et al. (2005) Early effects of salinity on water transport in Arabidopsis roots. Molecular and cellular features of aquaporin expression. Plant Physiol 139: 790-805. doi:10.1104/pp.105.065029. PubMed: 16183846. 16183846 PMC1255996

[47] Weihrauch D, Ziegler A, Siebers D, Towle DW (2002) Active ammonia excretion across the gills of the green shore crab Carcinus maenas: participation of Na+/K+-ATPase, V-type H+-ATPase and functional microtubules. J Exp Biol 205: 2765-2775. PubMed: 12177142. 12177142 10.1242/jeb.205.18.2765

[48] Henry RP, Garrelts EE, McCarty MM, Towle DW (2002) Differential induction of branchial carbonic anhydrase and Na+/K+ ATPase activity in the euryhaline crab, Carcinus maenas, in response to low salinity exposure. J Exp Zool 292: 595-603. doi:10.1002/jez.10075. PubMed: 12115925. 12115925

[49] Luquet CM, Weihrauch D, Senek M, Towle DW (2005) Induction of branchial ion transporter mRNA expression during acclimation to salinity change in the euryhaline crab Chasmagnathus granulatus. J Exp Biol 208: 3627-3636. doi:10.1242/jeb.01820. PubMed: 16169940. 16169940

[50] Jayasundara N, Towle DW, Weihrauch D, Spanings-Pierrot C (2007) Gill-specific transcriptional regulation of Na+/K+-ATPase α-subunit in the euryhaline shore crab Pachygrapsus marmoratus: sequence variants and promoter structure. J Exp Biol 210: 2070-2081. doi:10.1242/jeb.004309. PubMed: 17562880. 17562880

[51] Tsai J-R, Lin H-C (2007) V-type H+-ATPase and Na+,K+-ATPase in the gills of 13 euryhaline crabs during salinity acclimation. J Exp Biol 210: 620-627. doi:10.1242/jeb.02684. PubMed: 17267648. 17267648

[52] Drews G, Graszynski K (1987) The transepithelial potential difference in the gills of the fiddler crab, Uca tangeri: influence of some inhibitors. Journal of Comparative Physiology B 157: 345-353. doi:10.1007/BF00693361.

[53] Krippeit-Drews P, Drews G, Graszynski K (1989) Effects of ion substitution on the transepithelial potential difference of the gills of the fiddler crabUca tangeri: evidence for a H+-pump in the apical membrane. Journal of Comparative Physiology B, Biochemical, Systemic, and Environmental Physiology 159: 43-49. doi:10.1007/BF00692682.

[54] Onken H, Putzenlechner M (1995) A V-ATPase drives active, electrogenic and Na+-independent Cl-absorption across the gills of Eriocheir sinensis. Journal of Experimental Biology 198: 767-774. PubMed: 9318532. 9318532 10.1242/jeb.198.3.767

[55] Huong DTT, Yang WJ, Okuno A, Wilder MN (2001) Changes in free amino acids in the hemolymph of giant freshwater prawn Macrobrachium rosenbergii exposed to varying salinities: relationship to osmoregulatory ability. Comparative Biochemistry and Physiology A:_Molecular and Integrative Physiology 128: 317-326. doi:10.1016/S1095-6433(00)00310-X. PubMed: 11223393. 11223393

[56] Tan C, Choong K (1981) Effect of hyperosmotic stress on hemolymph protein, muscle ninhydrin-positive substances and free amino acids in< i> Macrobrachium rosenbergii</i>(de man). Comparative Biochemistry and Physiology Part A: Physiology 70: 485-489. doi:10.1016/0300-9629(81)92559-7.

[57] Dalla Via G (1986) Salinity responses of the juvenile penaeid shrimp< i> Penaeus japonicus</i >: I. Oxygen consumption and estimations of productivity. Aquaculture 55: 297-306

[58] Dalla Via G (1986) Salinity responses of the juvenile penaeid shrimp< i> Penaeus japonicus</i >: II. Free amino acids. Aquaculture 55: 307-316

[59] Lang MA (1987) Correlation between osmoregulation and cell volume regulation. Am J Physiol 252: R768-R773. PubMed: 3565606. 3565606 10.1152/ajpregu.1987.252.4.R768

[60] Dooley PC, Long BM, West JM (2000) Amino acids in haemolymph, single fibres and whole muscle from the claw of freshwater crayfish acclimated to different osmotic environments. Comp Biochem Physiol A Mol Integr Physiol 127: 155-165. doi:10.1016/S0305-0491(00)00247-9. PubMed: 11064283. 11064283

[61] Trachootham D, Lu W, Ogasawara MA, Valle NR-D, Huang P (2008) Redox regulation of cell survival. Antioxid Redox Signal 10: 1343-1374. PubMed: 18522489. 18522489 10.1089/ars.2007.1957 PMC2932530

[62] Mann M, Jensen ON (2003) Proteomic analysis of post-translational modifications. Nat Biotechnol 21: 255-261. doi:10.1038/nbt0303-255. PubMed: 12610572. 12610572

[63] Johnston M, Hutvagner G (2011) Posttranslational modification of Argonautes and their role in small RNA-mediated gene regulation. Silence 2: 1-4. doi:10.1186/1758-907X-2-1. PubMed: 21247442. 21943311 PMC3199228

[64] Huang QS, Yan JH, Tang JY, Tao YM, Xie XL et al. (2010) Cloning and tissue expressions of seven chitinase family genes in Litopenaeus vannamei. Fish Shellfish Immunol 29: 75-81. PubMed: 20202477. 20202477 10.1016/j.fsi.2010.02.014

[65] Salma U, Uddowla MH, Kim M, Kim JM, Kim BK et al. (2012) Five hepatopancreatic and one epidermal chitinases from a pandalid shrimp (Pandalopsis japonica): cloning and effects of eyestalk ablation on gene expression. Comp Biochem Physiol B Biochem Mol Biol 161: 197-207. doi:10.1016/j.cbpb.2011.11.005. PubMed: 22138334. 22138334

[66] Proespraiwong P, Tassanakajon A, Rimphanitchayakit V (2010) Chitinases from the black tiger shrimp Penaeus monodon: phylogenetics, expression and activities. Comp Biochem Physiol B Biochem Mol Biol 156: 86-96. doi:10.1016/j.cbpb.2010.02.007. PubMed: 20197105. 20197105

[67] Tan SH, Degnan BM, Lehnert SA (2000) The Penaeus monodon Chitinase 1 Gene Is Differentially Expressed in the Hepatopancreas During the Molt. Cycles - Mar Biotechnol (NY) 2: 126-135. 10.1007/s101269900016 10811951

[68] Mirzaei M, Pascovici D, Atwell BJ, Haynes PA (2012) Differential regulation of aquaporins, small GTPases and V-ATPases proteins in rice leaves subjected to drought stress and recovery. Proteomics 12: 864-877. doi:10.1002/pmic.201100389. PubMed: 22539437. 22539437

[69] Shikata Y, Rios A, Kawkitinarong K, DePaola N, Garcia JGN et al. (2005) Differential effects of shear stress and cyclic stretch on focal adhesion remodeling, site-specific FAK phosphorylation, and small GTPases in human lung endothelial cells. Exp Cell Res 304: 40-49. doi:10.1016/j.yexcr.2004.11.001. PubMed: 15707572. 15707572

[70] Marešová L, Vydarený T, Sychrová H (2012) Comparison of the influence of small GTPases Arl1 and Ypt6 on yeast cells' tolerance to various stress factors. FEMS Yeast Res 12: 332-340. doi:10.1111/j.1567-1364.2011.00780.x. PubMed: 22188384. 22188384

[71] Towle DW, Henry RP, Terwilliger NB (2011) Microarray-detected changes in gene expression in gills of green crabs (Carcinus maenas) upon dilution of environmental salinity. Comp Biochem Physiol Part D Genomics Proteomics 6: 115-125. PubMed: 21220218. 21220218 10.1016/j.cbd.2010.11.001

