# Supplementary figures and images for: Transcriptome Analysis of Portunus trituberculatus in Response to Salinity Stress Provides Insights into the Molecular Basis of Osmoregulation
Source: PLoS One. 2013 Dec 3;8(12):e82155. doi: 10.1371/journal.pone.0082155 (PMC3849447; doi:10.1371/journal.pone.0082155)

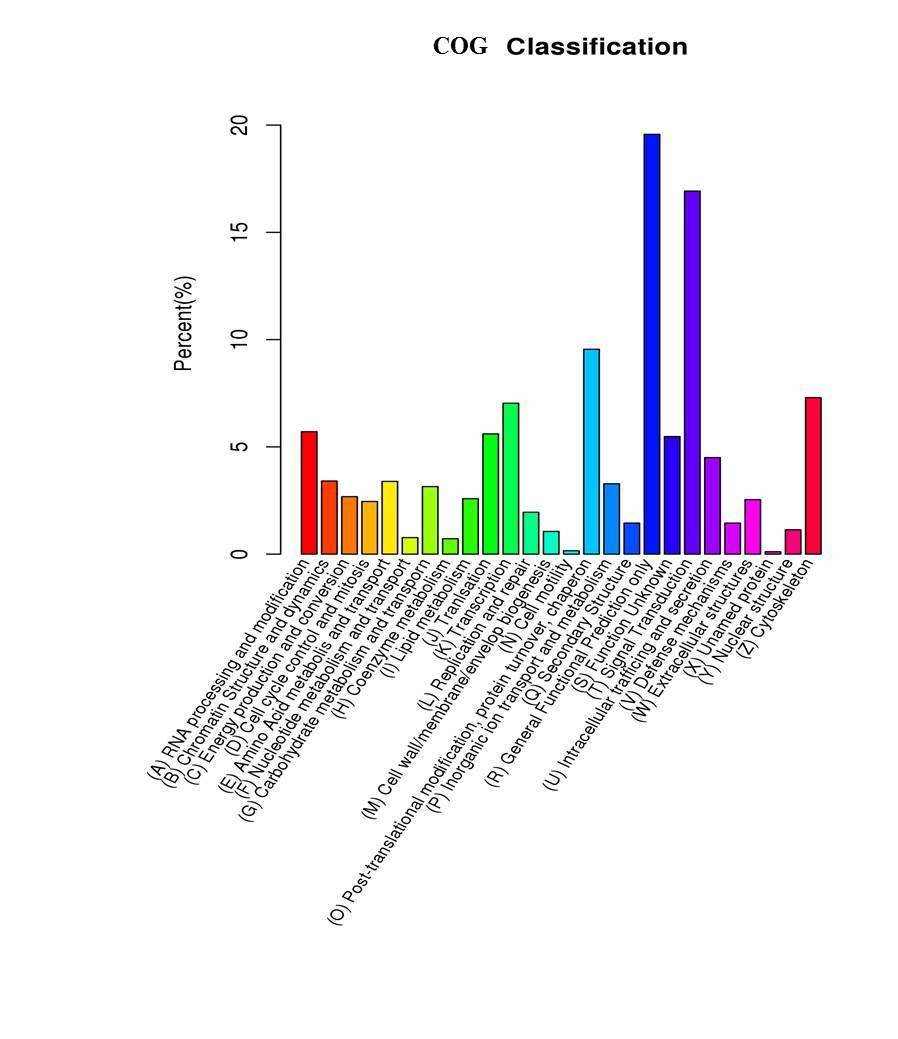

Supplement: Figure S1 — COG Classification of the unigenes. Possible functions of 11528 unigenes were classified and subdivided into 26 COG categories. (JPG) [file pone.0082155.s004.jpg]

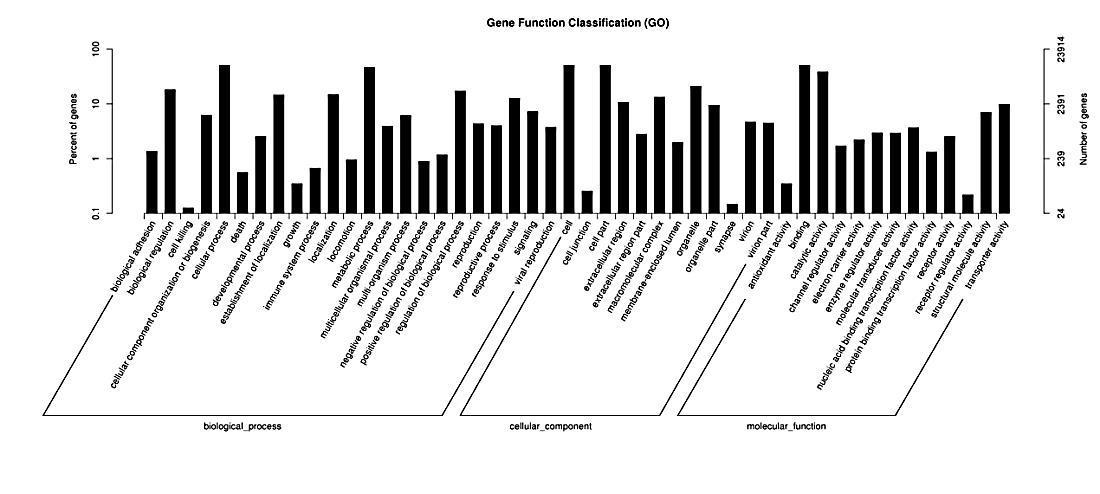

Supplement: Figure S2 — GO classification of all unigenes. Most unigenes canbe divided into three major categories, including biological process, cellular component, and molecular function. (JPG) [file pone.0082155.s005.jpg]

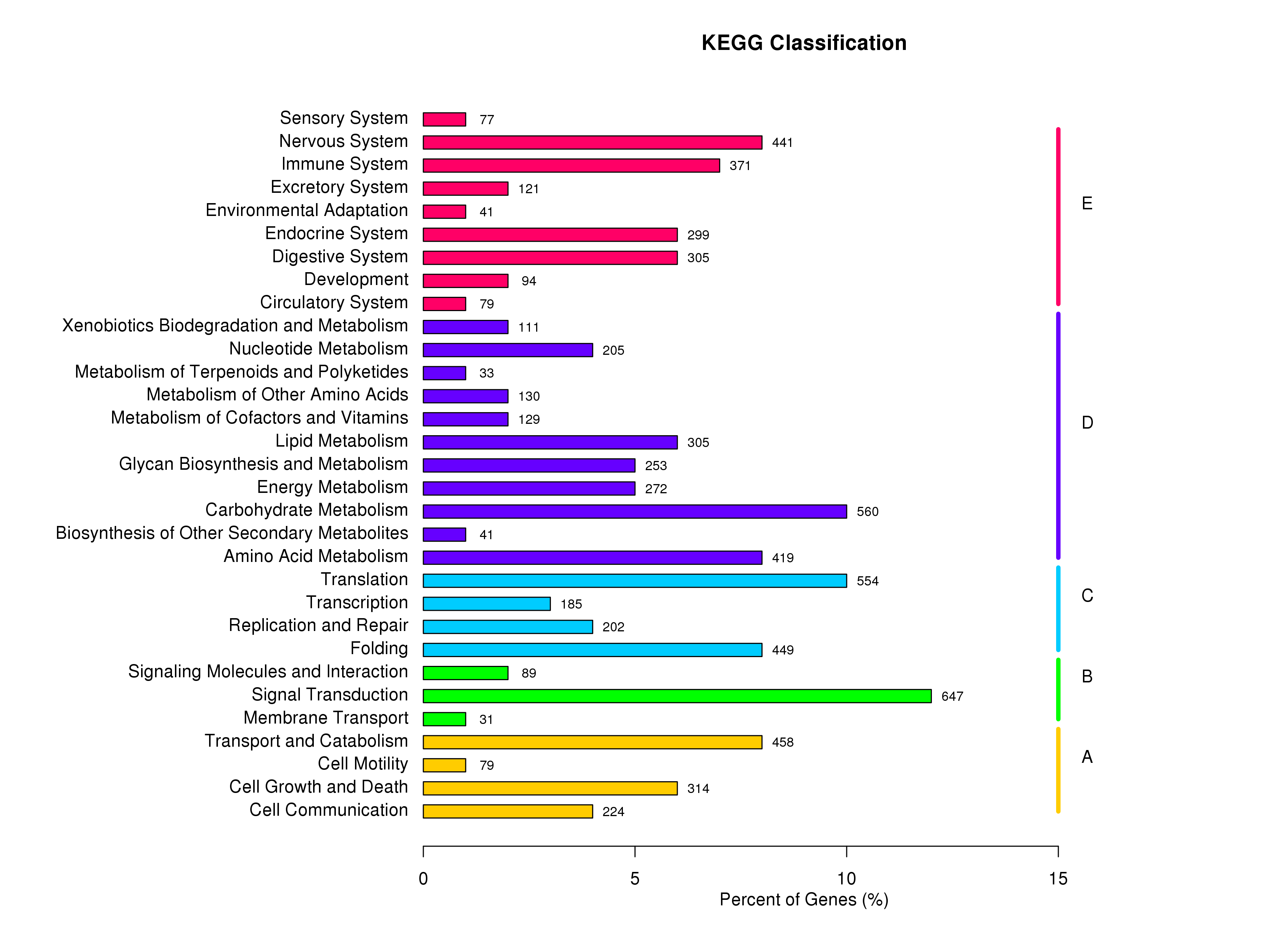

Supplement: Figure S3 — KEGG Classification of the unigenes. 5419 unigenes were assigned into 31 KEGG pathways.A, Cellular Processes; B, Enviromental Information Processing; C, Genetic Information Processing; D, Metabolism; E, Organismal Systems. (PNG) [file pone.0082155.s006.png]
